# Porcine dentin sialoprotein glycosylation and glycosaminoglycan attachments

**DOI:** 10.1186/1471-2091-12-6

**Published:** 2011-02-03

**Authors:** Yasuo Yamakoshi, Takatoshi Nagano, Jan CC Hu, Fumiko Yamakoshi, James P Simmer

**Affiliations:** 1 Department of Biologic and Materials Sciences, University of Michigan School of Dentistry, 1210 Eisenhower Place, Ann Arbor, MI, 48108, USA; 2 Department of Periodontics and Endodontics, School of Dental Medicine, Tsurumi University, 2-1-3 Tsurumi, Tsurumi-ku, Yokohama 230-8501, Japan

## Abstract

**Background:**

Dentin sialophosphoprotein (Dspp) is a multidomain, secreted protein that is critical for the formation of tooth dentin. Mutations in *DSPP *cause inherited dentin defects categorized as dentin dysplasia type II and dentinogenesis imperfecta type II and type III. Dentin sialoprotein (Dsp), the N-terminal domain of dentin sialophosphoprotein (Dspp), is a highly glycosylated proteoglycan, but little is known about the number, character, and attachment sites of its carbohydrate moieties.

**Results:**

To identify its carbohydrate attachment sites we isolated Dsp from developing porcine molars and digested it with endoproteinase Glu-C or pronase, fractionated the digestion products, identified fractions containing glycosylated peptides using a phenol sulfuric acid assay, and characterized the glycopeptides by N-terminal sequencing, amino acid analyses, or LC/MSMS. To determine the average number of sialic acid attachments per N-glycosylation, we digested Dsp with glycopeptidase A, labeled the released N-glycosylations with 2-aminobenzoic acid, and quantified the moles of released glycosylations by comparison to labeled standards of known concentration. Sialic acid was released by sialidase digestion and quantified by measuring β-NADH reduction of pyruvic acid, which was generated stoichiometrically from sialic acid by aldolase. To determine its forms, sialic acid released by sialidase digestion was labeled with 1,2-diamino-4,5-methyleneoxybenzene (DMB) and compared to a DMB-labeled sialic acid reference panel by RP-HPLC. To determine the composition of Dsp glycosaminoglycan (GAG) attachments, we digested Dsp with chondroitinase ABC and compared the chromotagraphic profiles of the released disaccharides to commercial standards. N-glycosylations were identified at Asn^37^, Asn^77^, Asn^136^, Asn^155^, Asn^161^, and Asn^176^. Dsp averages one sialic acid per N-glycosylation, which is always in the form of N-acetylneuraminic acid. O-glycosylations were tentatively assigned at Thr^200^, Thr^216 ^and Thr^316^. Porcine Dsp GAG attachments were found at Ser^238 ^and Ser^250 ^and were comprised of chondroitin 6-sulfate and chondroitin 4-sulfate in a ratio of 7 to 3, respectively.

**Conclusions:**

The distribution of porcine Dsp posttranslational modifications indicate that porcine Dsp has an N-terminal domain with at least six N-glycosylations and a C-terminal domain with two GAG attachments and at least two O-glycosylations.

## Background

Type I collagen and proteolytic cleavage products of dentin sialophosphoprotein (Dspp) are the predominant proteins in tooth dentin. Collagen constitutes about 90% of the dentin organic matrix [1], while Dspp-derived proteins make up more than half of the dentin noncollagenous proteins [2-5]. Genetic studies have demonstrated the importance of the genes encoding type I collagen (*COL1A1*, 17q21.31-q22; *COL1A2*, 7q22.1) and *DSPP *(4q21.3) for proper human dentin formation. Inherited dentin defects are classified as dentinogenesis imperfecta (DGI) types I, II, or III, or dentin dysplasia (DD) types I and II [6]. DGI type I is osteogenesis imperfecta with dentinogenesis imperfecta and is caused by mutations in *COL1A1 *and *COL1A2 *[7]. DD type II and DGI types II and type III are caused by mutations in *DSPP *[8-24]. There are other potential candidate genes for these disorders, but only *DSPP *mutations have been found in DD or DGI kindreds [25].

Dentin sialophosphoprotein (Dspp) is a chimeric protein that is cleaved by proteases into its component parts [26], which are considered to be two parts in rodents and three in pig. The order of the major domains of Dspp is Dsp-Dgp-Dpp, where Dsp is dentin sialoprotein, Dgp is dentin glycoprotein, and Dpp is dentin phosphoprotein. Dspp is expressed predominantly by odontoblasts [27], but is detected in bone at trace levels (0.25% of the level in dentin) [28]. Currently, the human expressed sequence tag database for *DSPP *expression (Hs.678914) shows zero DSPP transcripts out of 71,655 total mRNA transcripts characterized from bone.

*Dspp *is a member of the secretory calcium-binding phosphoprotein (SCPP) genes, which are involved in the mineralization of bone, dentin, and enamel [29]. The SCPP family initially arose from a single ancestral gene: *SPARCL1 *or secreted protein, acidic, cysteine-rich like 1 gene [30]. The common features of SCPP genes are few: their second exon encodes a short signal peptide (~15 amino acids in length) plus the first two amino acids of the secreted protein. The second coding exon generally encodes a phosphorylation site SXE/S(p) for Golgi casein kinase [30,31]. All of the introns are type 0, that is, the introns do not interrupt codons but are all placed between codon triplets. The SCPPs divide into two groups based upon their distinctive amino acid compositions. Acidic SCPPs are rich (over 25%) in glutamate, aspartate, and phosphoserine. Proline and glutamine-rich SCPPs are more than 20% proline and glutamine. *Dspp *belongs to the acidic group, which is also known as the dentin/bone group or the SIBLINGs, for small integrin-binding ligand, N-linked glycoproteins [32,33].

The second coding exon for *Dspp *(exon 3) is unusual for the SCPP family. In the pig, this exon encodes the protein segment from Val^3 ^to Gln^31^. None of the amino acids encoded by exon 3 are in the appropriate context for phosphorylation or glycosylation. Skipping exon 3 would not alter the reading frame, but human mutations that cause the skipping of exon 3 cause dominant negative effects that result in dentin malformations, possibly due to failure to recognize or cleave the signal peptide [20]. All six of the reported intronic mutations in *DSPP *that cause inherited dentin defects are at the end of intron 2 [9,20,24] or beginning of intron 3 [12,14,17,18]. Perhaps exon 3 was acquired by exon capture after the *Dspp *ancestral gene duplicated from another SCPP gene and is not descended from the short prototypical second coding exon that encodes a serine that gets phosphorylated.

Exon 4 encodes the bulk of dentin sialoprotein (Dsp), from Asp^32 ^to Lys^368 ^[34]. Previously we isolated and characterized a porcine "Dsp only" transcript. This mRNA transcript terminated at a polyadenylation/cleavage site in intron 4 [35]. The porcine Dsp cleavage product (Ile^1 ^to Arg^376^) of Dspp is a highly glycosylated proteoglycan that forms covalent dimers [36]. The Dgp cleavage product (Ser^377 ^to Gly^457^) of Dspp is encoded by the 5' part of exon 5. Dgp, which has so far only been described in pig, has a single glycosylation (Asn^382^) and four phosphoserines (Ser^438^, Ser^440^, Ser^442^, Ser^447^) that are in a highly conserved region near its carboxyl-terminus [37]. Dentin phosphoprotein (Dpp) is the highly phosphorylated carboxyl-terminal domain of Dspp, and is encoded by the rest of exon 5. The coding region of Dpp varies in length among different individuals without affecting function [20,38]. Astacin proteases, such as Bmp-1 cleave Dpp from Dspp [39-41], while Mmp-20 separates Dsp and Dgp [42].

Dentin sialoprotein (Dsp) was previously thought to be only a minor component of dentin, perhaps only 10% as abundant as dentin phosphoprotein (Dpp) [43,44], and later thought to be a nonfunctional pro-domain that was removed by proteases to activate Dpp [45]. Recently, however it has been determined that Dsp and Dpp are expressed in stoichiometric amounts as the N-terminal and C-terminal domains of Dspp [26] and are found in equal molar amounts in dentin [38,46]. *Dspp *null mice show severe dentin malformations that resemble those observed in human DGI-III [47]. Transgenic expression of the Dsp domain in the *Dspp *null background partially, but substantially, restores the dentin phenotype [48]. The widening of predentin observed in the *Dspp *knockout as well as the reduced volume of mineralized dentin are recovered, indicating that both Dsp and Dpp are necessary for normal dentin mineralization. The mechanisms through which Dsp contributes to dentin formation are unknown. Characterization of its posttranslational modifications (PTMs) is an important step in understanding its function.

## Results

### Characterization of Dsp carbohydrate attachment sites

We previously showed that porcine Dsp is a proteoglycan [36] and that porcine dentin extracts contain the intact Dsp (the N-terminal domain of Dspp), as well as lower molecular weight (LMW) cleavage products of Dsp [42]. It was also known that Dsp has glycosylations rich in sialic acid [34] and that porcine Dsp has eight potential N-glycosylation sites based upon analysis of its primary amino acid sequence [35]. To identify attachment sites and to better characterize the glycosaminoglycan (GAG) and other carbohydrate attachments in porcine Dsp, we first targeted the small glycosylated Dsp cleavage products (LMW-Dsp) from the N-terminal region of Dsp. As none of the dentin extracts are perfectly homogeneous with respect to their contents, we refer to each extract that we characterized using a notation that reflects the extraction and fractionation steps that produced it (see methods). Routine characterizations of these fractions (SDS-PAGE and Western blots) are provided in additional files online.

**Figure 1:**
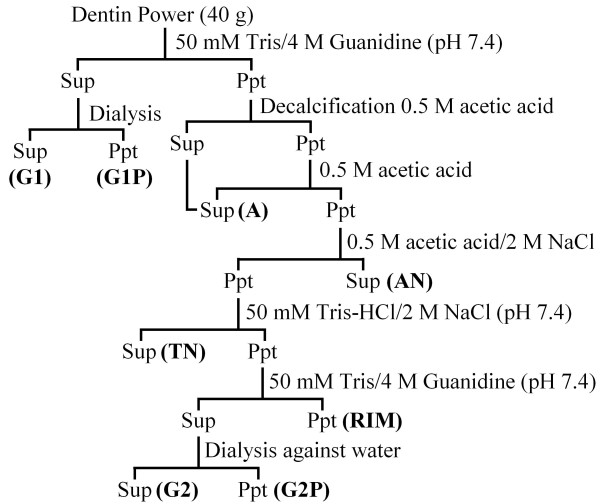
**Dentin power extraction diagram**. The extracts, in bold, are the first guanidine extract (G1), first guanidine pellet (G1P), acid (A), acid salt (AN), Tris salt (TN), residual insoluble material (RIM), second guanidine extract (G2), and the second guanidine pellet (G2P). Selected extractions were fractionated to isolate Dspp cleavage products. The AN extract was fractionated by size exclusion (S) and RP-HPLC (R) to yield HMW Dsp in fractions ANS1/2-R3, ANS1/2-R4, and ANS1/2-R5. The A extract was fractionated by size exclusion and RP-HPLC to yield LMW Dsp (AS2R-g). Chondroitinase activity was detected in the second ion exchange (Q) fraction of the G2 extact (G2Q2).

**Figure 2:**
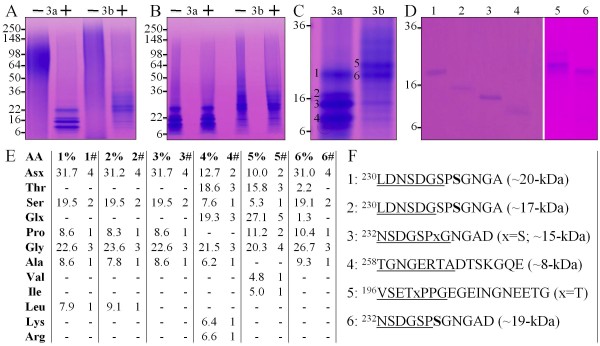
**Characterization of small, highly glycosylated Dsp peptides**. ***A: ***4-20% Tris/glycine gel stained with stains-all showing fraction ANS1/2R3-a and -b before (-) and after (+) digestion with chondroitinase ABC. The stains-all positive proteoglycan smears coalesced into a group of protein bands having apparent molecular weights ranging from 8 to 20-kDa. ***B: ***4-20% Tris/glycine gel stained with stains-all showing fraction ANS1/2R3-a and -b without (-) and with (+) the reducing agent β-mercaptoethanol. The mobilities of the bands did not change indicating the absence of disulfide bridges. ***C: ***4-20% Tris/glycine gel stained with stains-all showing the bands from ANS1/2R3-a (3a) and ANS1/2R3-b (3b) following chondroitinase ABC digestion that were excised from the gel and characterized by amino acid analysis and N-terminal sequencing. ***D: ***18% Tris/glycine gel stained with stains-all showing the bands following electroelution. ***E: ***Results of amino acid analyses showing the percentage (%) of each amino acid in the 6 peptides and the deduced numbers (#) of each amino acid. The percentages were converted into number of amino acids by dividing all of the percentages by the lowest meaningful percentage, which corresponded to the percentage of a single amino acid. ***F: ***The complete sequences of each peptide. The Edman sequences are underlined with blank cycles indicated by an "x".

Demineralized dentin powder was subjected to a series of extraction procedures. A flow chart of the extractions is provided in Figure 1. Simple analyses of the extracts (SDS-PAGE stained with CBB and stains-all and a Dsp immunoblot) are shown in Additional file 1. Intact Dsp proteoglycan and its cleavage products containing GAG attachments segregated in the acid/NaCl or "AN" extract, which was further fractionated by size exclusion chromatography. Dsp degradation products containing the GAG attachments displayed a stains-all positive smear migrating between 50- to 100-kDa on SDS-PAGE, which were collected in the third (ANS1/2-R3) of five RP-HPLC fractions (Additional file 2). ANS1/2-R3 was resolved into two final fractions by repeating the RP-HPLC run. These two fractions (ANS1/2-R3a and ANS1/2-R3b) were digested with chondroitinase ABC to degrade their GAG attachments, which converted the proteoglycan smears on SDS-PAGE into small peptides (Figure 2A) that contained no disulfide bridges, as their mobilities were not affected by the presence or absence of β-mercaptoethanol (Figure 2B). Chondroitinase ABC digestion converted ANS1/2-R3a into four peptides and ANS1/2-R3b into two peptides having apparent molecular weights ranging from 8- to 25-kDa (Figure 2C). These six peptides were excised and eluted from the gel (Figure 2D) and then characterized by N-terminal sequencing and by amino acid analysis (Figure 2E). The N-terminal sequence and amino acid compositions allowed the complete amino acid sequences of the six peptides to be deduced (Figure 2F).

Peptides 1, 2, 3 and 6 contained the putative GAG attachment site at Ser^238 ^and the amino acid compositions for each of these proteins showed one fewer serine than expected. N-terminal sequence for peptide 3 gave a blank cylce for Ser^238^. The other N-terminal sequences did not reach Ser^238^, but the full-length of each peptide could be inferred from its amino acid composition. A blank cycle strongly suggests the presence of a posttranslational modification, but additional evidence is generally required to support this conclusion. In addition, the four peptides containing Ser^238 ^were only 11 or 12 amino acids in length, but migrated at 15-, 17-, 19- and 20-kDa on SDS-PAGE, suggesting they retained variable lengths of GAG attachment following the chondroitinase ABC digestion. From these data we can safely conclude that Ser^238 ^is a GAG attachment site; however, no information from this experiment was gained on the other putative GAG attachment sites on porcine Dsp (at Ser^220 ^and Ser^250^) [36]. No conclusions could be made from the analyses of peptides 4 and 5. A blank cycle was observed at Thr^200^, but the amino acid composition suggested that this position was a threonine, and therefore not necessarily modified.

**Figure 3:**
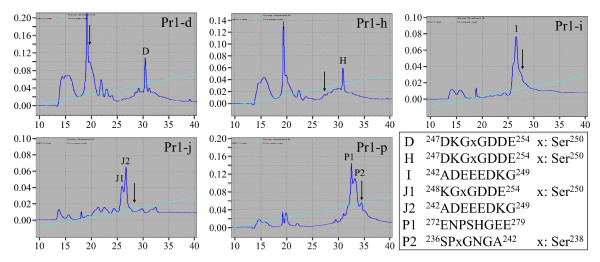
**Characterization of ANS1/2-R3 pronase digestion products with altered retention times following chondroitinase ABC digestion**. RP-HPLC chromatograms (Abs^230 ^v. min) ANS1/2R3Pr1-d, -h, -i, -j, and -p following chondroitinase ABC digestion. Arrows indicate retention times of the main peak prior to chondroitinase ABC digestion. N-terminal sequences determined for the contents of each labeled peak (D, H, I, J1, J2, P1 and P2) are shown on the lower right. Blank cycles, indicated by an "x", identified Ser^238 ^and Ser^250 ^as modified residues.

To gain additional information on posttranslational modifications in the GAG attachment region of Dsp, fraction ANS1/2-R3 was digested with pronase. Larger digestion products from the pronase digestion were recovered by size exclusion chromatography in the first eluted fraction (ANS1/2R3-Pr1) (Additional file 3). ANS1/2R3-Pr1 resolved into 26 fractions by RP-HPLC and each was assayed for the presence of glycosylations using the phenol-sulfuric acid assay. Glycosylated peptides were detected in eight of the RP-HPLC fractions: ANS1/2R3Pr1-d, -e, -h through -l, and -p (Additional file 3). To identify peptides with GAG attachments, the eight glycosylated fractions were digested with chondroitinase ABC. The peptides were separated from the digested carbohydrate by ethanol precipitation and each supernatant (containing the liberated GAG chains) was analyzed by phenol sulfuric acid assay. Fractions positive for glycan attachments (-d, -h, -i, -j, and -p) were rechromatographed using the same C-18 column that was used prior to the chondroitinase ABC digestion. Rechromatographed fractions showing a shift in their retention times relative to same sample prior to chondroitinase ABC digestion were characterized by N-terminal sequencing (Figure 3). One peptide (ANS1/2R3Pr1p-P2) gave a blank cycle for Ser^238^, while three peptides (ANS1/2R3Pr1d-D, ANS1/2R3Pr1h-H, and ANS1/2R3Pr1j-J1) gave blank cycles for Ser^250^. These data demonstrate that Ser^238 ^and Ser^250 ^are the GAG attachment sites for porcine Dsp. The N-terminal sequences of the peptides in fractions ANS1/2R3Pr1-k and ANS1/2R3Pr1-l, which were negative for GAG chains based upon the phenol sulfuric acid assay, were ^211^EETGVxSGGSGA and ^311^TDGDNxSK, respectively. The blank cycles for Thr^216 ^and Thr^316 ^suggest these residues are modified, but these sequences also show that Ser^220 ^and Asn^315 ^(which did not give blank cycles) are not modified, despite their being in appropriate contexts for GAG or N-glycosylation attachments, respectively.

**Figure 4:**
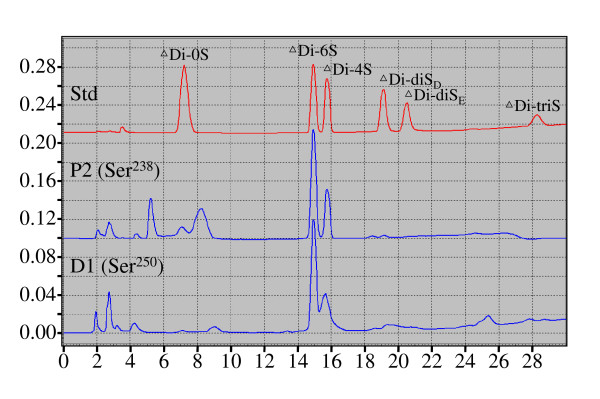
**Characterization of disaccharides attached at Ser^238 ^and Ser^250^**. Supelcosil LC-NH_2 _chromatograms (Abs^232 ^v. min) of GAG products released from ANS1/2R3-Prp (Ser^238^) and ANS1/2R3-Prd (Ser^250^) by chondroitinase ABC digestion compared to GAG standards (ΔDi-0S; ΔDi-6S; ΔDi-4S; ΔDi-diS_D_; ΔDi-diS_E_; ΔDi-triS). Based upon their retention times, the GAG attachments are observed to contain chondroitin 6-sulfate (ΔDi-6S) and chondroitin 4-sulfate (ΔDi-4S). Integration of the areas under the peaks show the ratio of C6 S to C4 S is 7 to 3.

Having established that Ser^238 ^and Ser^250^, but not Ser^220^, have GAG attachments, we then characterized the nature of the disaccharide attachments at these two GAG attachment sites. Peptides ANS1/2R3Pr1d-D (with the GAG attached at Ser^250^) and ANS1/2R3Pr1p-P2 (with the GAG attached at Ser^238^) were digested by chondroitinase ABC. The released carbohydrates were obtained from the supernatant following ethanol precipitation of the peptide, and were characterized by their retention times relative to those of disaccharide standards on NP-HPLC (Figure 4). This determined that both GAG attachment sites contained chondroitin-6-sulfate (C6S) and chondroitin-4-sulfate (C4S), and integration of the areas under their chromatogram peaks determined that C6 S and C4 S are present in a ratio 7 to 3, respectively.

### Characterization of R4 Pronase Digestion Products

**Figure 5:**
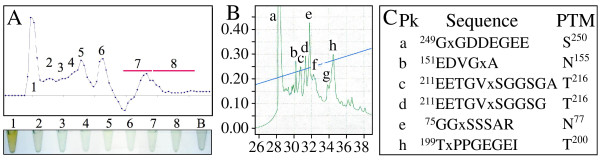
**Characterization of glycosylated pronase digestion products of ANS1/2-R4**. ***A: ***Size exclusion chromatogram (Abs^220 ^v. min) of ANS1/2-R4 digested with pronase (top); tubes showing results of phenol sulfuric acid assay performed on each size exclusion fraction (bottom; B is "blank" for negative control). ***B: ***RP-HPLC chromatogram (Abs^220 ^v. min) of digestion products in ANS1/2R4-Pr1 showing the 8 fractions (a-h) collected. ***C: ***N-terminal sequences determined for the contents of each fraction peak (Pk). Blank cycles (x) indicate modified residues.

Previously we showed that intact Dsp (Dspp-derived protein that lacks the Dgp and Dpp regions) is in fraction ANS1/2-R4 [42]. This R4 fraction (5 mg) was digested with pronase, and glycosylated peptides were collected in the first peak by size exclusion chromatography (Figure 5A). These peptides resolved into eight peaks by RP-HPLC (Figure 5B). N-terminal sequencing of these peptides gave blank cycles for Asn^77^, Asn^155^, Thr^200^, Thr^216^, and Ser^250^.

### Characterization of Dsp N-linked glycosylation sites

**Figure 6:**
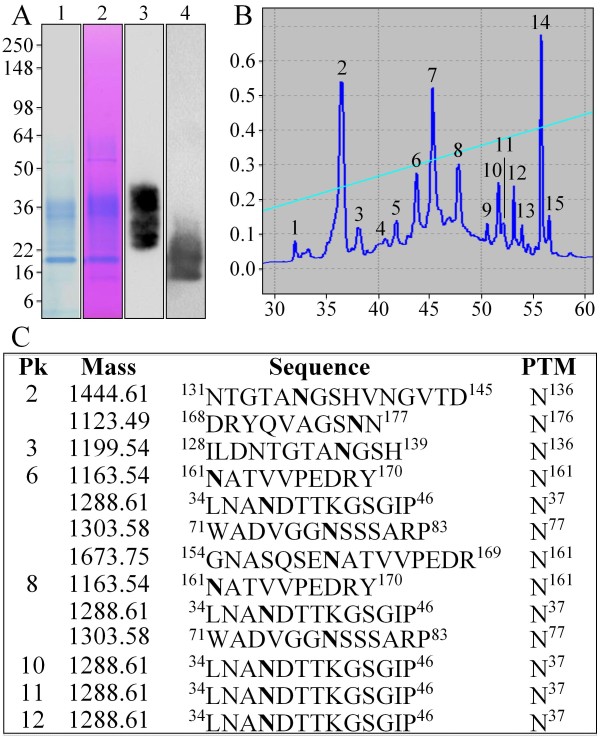
**Characterization of glycosylated endoproteinase Glu-C digestion products of LMW Dsp**. ***A: ***Contents of fraction AS2R-g characterized by SDS-PAGE stained with CBB (1), stained with stains-all (2); by Western blotting using the Dsp antibody (3) or Dgp antibody (4). ***B: ***RP-HPLC chromatogram (Abs^220 ^v. min) of endoproteinase Glu-C digestion products of AS2R-g. ***C: ***Table showing the results of mass spectrometric (LC/MSMS) analyses of RP-HPLC fractions following glycopeptidase digestion. Asparagines (N) in the appropriate context for N-glycosylation that were also found to be 1 Da smaller than expected (due to deamidation) are indicated in bold and listed as the posttranslational modification (PTM) site on the right.

Previously we showed by Western blot analyses that low molecular weight (LMW) peptides from the N-terminal region of Dsp can be found in the acid or "A" extract [42]. To identify glycosylated amino acids in these peptides, the A extract was fractionated by size exclusion chromatography followed by RP-HPLC (Additional file 4). The LMW Dsp components in fraction AS2R-g (Figure 6A) were digested with endoproteinase Glu-C (V8 endoproteinase) and the digest was fractionated by RP-HPLC (Figure 6B). Fifteen peaks were observed and each of these fractions was digested with glycopeptidase A. Following the N-deglycosylation, each sample was rechromatographed on the same C-18 column to demonstrate that deglycosylation of the glycopeptide had altered its retention time. A shift in retention time on the column following deglycosylation was accepted as evidence that the original peptide was glycosylated. Fractions corresponding to the main chromatographic peaks that were not present before the glycopeptidase A digestion (AS2R-g peaks 2, 3, 6, 8, 10, 11 and 12) were characterized by LC/MSMS analysis (Figure 6C). Five N-glycosylation sites were identified: Asn^37 ^(NDT; peaks 6, 8, 10, 11, 12), Asn^77 ^(NSS; peaks 6, 8), Asn^136 ^(NGS; peaks 2 and 3), Asn^155 ^(NAS; peak 6), Asn^161 ^(NAT; peaks 6, 8), and Asn^176 ^(NNS; peak 2).

### Analysis of Sialic Acid in Dsp

It was known early on that Dsp glycosylations are rich in sialic acid, which was the basis for its designation as dentin sialoprotein [34]. Sialic acid refers to N-acetylneuraminic acid (Neu5Ac) and its derivatives. To determine the amount of N-glycosylations in each Dspp fraction, we released the N-glycosylation with glycopeptidase A and chemically attached a fluorescent label. One fluorescent label was added per N-glycosylation. The amount of N-glycosylation in each Dsp fraction was determined by running an aliquot of labeled glycosylation over an SE-HPLC column and then comparing the areas under the chromatographic peaks to that of a commercial A2 glycan standard of known amount and fluorescence (Additional file 5). To quantify the average number of sialic acid attachments per N-glycosylation, we digested LMW-Dsp (containing N-terminal fragments of Dsp) and R5-Dsp (containing Dspp minus the Dpp domain) fractions with sialidase. The released sialic acid was digested with aldolase and the product (pyruvate) and lactic dehydrogenase were used to oxidize β-NADH to β-NAD, which was measured spectrophotometrically. The same series of reactions were conducted on a commercial standard (bovine fetuin). The results showed that there were, on average, 1.3 sialic acids per N-glycosylation in the N-terminal region of Dsp, and about 0.8 molecules of sialic acid, on average, per N-glycosylation in Dspp (Additional file 5).

To identify which forms of sialic acid were present on the N-linked glycosylations, N-glycosylations were released from the various Dsp fractions (LMW-Dsp, R3-Dsp, R4-Dsp, R5-Dsp), given a fluorescent label, and purified by size exclusion chromatography. Sialic acid was released from these glycosylations by sialidase digestion, labeled, and then passed through a RP-HPLC column. By comparing the retention times of the labeled sialic acid from the Dsp fractions to a similarly-labeled sialic acid reference panel it was demonstrated that all of the sialic acid attached to Dsp N-glycosylations are N-acetylneuraminic acid (Neu5Ac or NANA), which is the predominant form of sialic acid in mammalian cells (Additional file 5).

## Discussion

**Figure 7:**
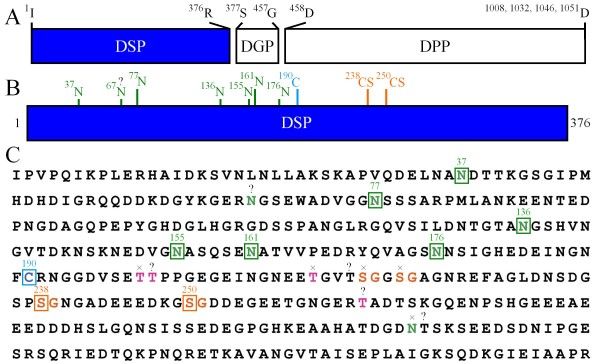
**Porcine Dsp posttranslational modification sites**. ***A: ***Diagram showing the three regions (Dsp, Dgp, and Dpp) of porcine Dspp. ***B: ***Diagram showing N and O-linked glycosylation sites identified in this study. Among the eight asparagines in the appropriate context for glycosylation, six were determined to be glycosylated (Asn^37^, Asn^77^, Asn^136^, Asn^155^, Asn^161^, Asn^176^). The glycosylation status Asn^67 ^was not determined. Asn^315 ^was shown to not be glycosylated, possibly due to an O-glycosylation of Thr^316^. In porcine Dsp, it was shown that the N-glycosylations contain, on average, one sialic acid, in the form of N-acetylneuraminic acid, per N-glycosylation. Ser^238 ^and Ser^250 ^were determined to be GAG attachment sites, with the GAG attachments being comprised of chondroitin 6-sulfate and chondroitin 4-sulfate in a ratio of 7 to 3. O-glycosylations were suggested by blank cycles at Thr^200^, Thr^216^, and Thr^316 ^in samples that were positive for glycosylation by the phenol sulfuric acid assay and eluted early from a size-exclusion column, but no direct evidence for O-glycosylation was obtained and the conclusion that these residues are glycosylated should be considered tentative.

In this study we identified six N-linked and three tentative O-linked glycosylation and the two GAG attachment sites on porcine Dsp (Figure 7). The two GAG attachment sites are particularly interesting. Xylosyltransferase I (*XYLT1*; 16p13.1) and xylosyltransferase II (*XYLT2*; 17q21.3) catalyze the initial step in glycosaminoglycan biosynthesis and determine if a site will have a GAG attachment [49]. Experimentally determined GAG attachment sites identify the range of amino acid contexts that are recognized by these enzymes and provide the data needed to improve the prediction algorythms for GAG attachment sites.

The two GAG attachment sites are in the most highly conserved area of Dsp (Additional file 6). The context for the second site (Ser^250^; DEEEDKG**S**GDDEGEE) contains a GSG-motif combined with a high number of surrounding acidic amino acids, so this sequence would be predicted to be a good acceptor for a xylosylation reaction and result in a high degree of xylosylation catalyzed by both XT-isoforms [50].

The first site (Ser^238^, DNSDGSP**S**GNGADEEED) has two acidic amino acids on the N-terminal side and a series of acidic amino acids starting at positions +5 or +6, which are positive features. However, the proline in the -1 position is thought to have an inhibitory effect on the glycosylation reaction [51]. Despite this, our results indicate that Ser^238 ^is a GAG attachment site. Perhaps the high conservation of the surrounding amino acids helps ensure its recognition as a target for glycosylation. Recently, it was shown that altering the potential GAG attachment sites sequences in rat Dsp diminished the apparent size of the recombinant protein on Western blots, indicating that both of the GAG attachments sites identified in this study have GAG attachments in rat Dsp [46].

It is clear from our analyses that the repeating disaccharide units attached to Ser^238 ^and Ser^250 ^are chondroitin 6-sulfate and chondroitin 4-sulfate, with C6 S predominating. Based upon antibody analyses, we previously detected only C6 S attached to porcine Dsp [36]. Dentin matrix protein 1 (Dmp1) is a bone and dentin proteoglycan that plays a role in phosphate homeostasis [52]. In rat, the predominant disaccharide unit on Dsp and Dmp1 is C4 S [53,54].

The role of Dsp GAG chains in dentin formation is largely unexplored. Proteoglycans such as decorin are known to regulate collagen fibrillogenesis [55,56]. GAGs are thought to interact directly with dentin collagen fibrils, as an electrondense substance that can be removed from dentin collagen fibrils by chondroitinase treatment [57]. Dsp is the most abundant proteoglycan in dentin and is therefore likely to interact with collagen. Chondroitinase activity in the dentin matrix might regulate interactions between the Dsp GAG chains and collagen.

Besides having two GAG attachments, porcine Dsp has at least six N-glycosylations. All of these sites are on the N-terminal side of the lone cysteine (Cys^190^) that covalently links two Dsp proteins into a dimer. Although a high degree of N-glycosylation is a characteristic feature of Dsp, the individual glycosylation sites are not well conserved. The N-linked glycosylations on porcine Dsp average about one sialic acid per N-glycosylation. Previously it was shown that rat Dsp contained 29.6% carbohydrate including about 9% sialic acid [34]. Sialic acid attachments can serve a wide range of functions [58]. Sialic acid moities can be ligands for other molecules, such as lectins. Perhaps Dsp could form a bridge between molecules that interact with sialic acid and others that interact with O-glycosylations or GAG attachments.

The two O-glycosylations and two GAG attachments are all on the C-terminal side of Cys^190^. Ser^238^, Ser^250^, and Thr^216 ^are perfectly conserved, whereas Thr^316 ^is conserved in all known Dsp sequences except the mouse. Thus the O-glycosylations and glycosaminoglycan attachments are structural elements that are likely to be essential for Dsp function. Three predicted Golgi casein kinase phosphorylation sites (Ser^294^, Ser^296 ^and Ser^319^) are all found in the C-terminal side of Dsp and are perfectly conserved.

The map of porcine Dsp posttranslational modifications suggests that Dsp is divided into two domains that are separated by the lone cysteine (Cys^190^) that covalently links two Dsp proteins. The N-terminal side is rich in sialidated N-glycosylations; the C-terminal side contains the GAGs, O-glycosylations, and phosphorylated serines.

The Dsp domain of Dspp is not highly phosphorylated (only 3 predicted sites). In contrast, the small Dgp domain has four perfectly conserved sites, while Dpp has well over a hundred. The Dsp domain is not highly conserved. Only 73 of 377 (19%) if its amino acids are perfectly conserved among the seven mammalian Dsp sequences in GenBank, whereas 28/81 (35%) of Dgp is perfectly conserved. The most highly conserved regions of Dsp and Dgp are in areas of posttranslational modifications, highlighting their importance for function. Dsp is important for the onset of dentin mineralization, and future studies will investigate the importance PTMs in carrying out these functions.

## Conclusions

Dentin sialoprotein (Dsp), the N-terminal domain of dentin sialophosphoprotein (Dspp) was isolated from developing porcine molars and its posttranslational modification were characterized. Porcine Dsp has at least 6 N-linked glycosylations (Asn^37^, Asn^77^, Asn^136^, Asn^155^, Asn^161^, and Asn^176^) that average one sialic acid each, all in the form of N-acetylneuraminic acid. Porcine Dsp has exactly two glycosaminoglycan attachments (Ser^238 ^and Ser^250^) that are comprised of chondroitin 6-sulfate and chondroitin 4-sulfate in a ratio of 7 to 3. Three O-linked glycosylations in Dsp were tentatively assigned (Thr^200^, Thr^216^, and Thr^316^). Glycosylations divide porcine Dsp into a heavily N-glycosylated, sialidated N-terminal domain and a C-terminal domain with two GAG attachments, three O-glycosylations, and three potential phosphoserines. The two Dsp domains are separated by the lone cysteine (Cys^190^) that connects two Dsp chains into a covalent dimer. Knowledge of Dsp structure is an important starting point to understanding the how Dsp serves to initiate mineralization of dentin.

## Methods

All experimental procedures involving the use of animals were reviewed and approved by the Institutional Animal Care and Use Program at the University of Michigan.

### Extraction of proteins from dentin powder

Maxillary and mandibular second molar tooth germs were extracted from 6 month old pigs and the soft tissue discarded. Enamel was removed by scraping with a curette and the remaining hard tissue was ground to a powder. All extraction steps were carried out at 4°C or on ice. The Protease Inhibitor Cocktail Set III (1 mM AEBSF, 0.8 μM aprotinin, 50 μM bestatin, 15 μM E-64, 20 μM leupeptin, and 10 μM pepstatin) (Calbiochem, San Diego, CA) and 1 mM 1,10-phenanthroline (Sigma-Aldrich, St. Louis, MO) were added into the buffer during the extraction. The extraction scheme is outlined in Figure 1. Forty grams of dentin powder (the amount obtained from 32 molars) was homogenized at 4°C in 50 mM Tris-HCl/4 M guanidine buffer (pH 7.4) containing protease inhibitors. Insoluble material was pelleted by centrifugation and the supernatant was designated the first guanidine (G1) extract. The guanidine pellet was dialyzed against 4 L of 0.5 M acetic acid containing protease inhibitors until the tooth mineral has fully dissolved. The dialysis bag contents were centrifuged, and the supernatant was designated the acid (A) extract. The pellet was extracted with 0.5 M acetic acid/2 M NaCl and the supernatant was designated the acid salt (AN) extract. The AN pellet was extracted with 50 mM Tris-HCl/2 M NaCl buffer (pH 7.4) and the supernatant designated the Tris salt (TN) extract. The TN pellet was extracted with 50 mM Tris-HCl/4 M guanidine buffer (pH 7.4). Residual insoluble material (RIM) was pelleted by centrifugation. The guanidine supernatant was dialyzed against water. Following centrifugation, the pelleted material that had precipitated during dialysis was designated the second guanidine pellet (G2P), while the supernatant was designated the second guanidine extract (G2).

### Purification of Dspp-derived proteins

Western blot analyses using a polyclonal antibody raised against recombinant porcine Dsp [36] showed that low molecular weight (LMW) Dsp components were in the A extract, while high molecular weight (HMW) Dsp components were in the AN extract (Additional file 1). The AN and A extracts were fractionated by size exclusion chromatography and the fraction containing Dsp positive material (based upon Western blot analyses) was further fractionated by reversed phase-high performance liquid chromatography (RP-HPLC). For the AN extract, the HMW Dsp components were found in the first two chromatographic peaks (AN-S1 and AN-S2), which were combined and resolved into five fractions by RP-HPLC [42]. The last three fractions to elute, designated ANS1/2-R3, ANS1/2-R4, and ANS1/2-R5, were comprised of successively larger stains-all positive smears of Dsp proteoglycan, respectively (Additional file 2). The LMW Dsp components in the A extract were found in the second (S2) of three fractions produced by size exclusion chromatography. S2 resolved into 14 fractions (designated a through n) on a POROS R2 column (4.6 mmID × 10 cm), equilibrated with 0.05% TFA and eluted with a linear acetonitrile gradient containing 0.05% TFA at a flow rate of 1.0 mL/min at room temperature. The LMW Dsp components were identified in fraction g (AS2R-g; Additional file 4).

To identify glycosylated peptides in porcine Dsp, the LMW and HMW Dsp components were digested with endoproteinase Glu-C or pronase and the glycopeptide products characterized. Endoproteinase Glu-C (Staphylococcus aureus Protease V8) is a serine proteinase that selectively cleaves peptide bonds C-terminal to acidic residues with a strong preference for glutamic over aspartic acid residues [59]. Pronase is a cocktail of proteolytic enzymes that degrades all but short glycopeptides that are protected from cleavage by bulky carbohydrate chains.

### Chondroitinase ABC digestion and characterization ANS1/2-R3

Fraction ANS1/2-R3, which produced a stains-all positive smear on sodium dodecyl sulfate-polyacrylamide gel electrophoresis (SDS-PAGE), was resolved into two parts (ANS1/S2-R3a and ANS1/S2-R3b) by RP-HPLC rechromatography (Additional file 2), lyophilized, and 200 μg of each part was resuspended in 40 mM Tris-HCl/40 mM sodium acetate buffer (pH 8.0) and incubated with 0.2 units of chondroitinase ABC protease free (Associate of Cape Cod Inc., East Falmouth, MA) at 37°C for 35 h. The digests resolved into six stains-all positive bands on SDS-PAGE, which were excised from the gel. Each gel slice was transferred to a DTube Dialyzer (midi size) (Novagen/EMD Chemicals, Inc., Gibbstown, NJ) and the protein electroeluted with 25 mM Tris/250 mM Tricine/0.025% SDS buffer (pH 8.5) at 150 volts for 3 h. Each eluate was precipitated with 20% trichloroacetic acid, incubated in acetone overnight at -80°C and centrifuged at 4°C for 30 min at 14,000 × g. The supernatant was decanted, the pellet was dried under a hood, and was later characterized by Edman sequencing.

### Characterization of ANS1/2-R3 following pronase digestion

Purified ANS1/2-R3 (33 mg) in 50 mM Tris-HCl buffer (pH 8.0) was incubated with 0.2 mg of pronase at 37°C for 20 h. The digestion products were fractionated by size exclusion chromatography using a Sephadex G-15 column (1.6 cm × 60 cm). The first eluted fraction of the pronase digest was positive for oligosaccharide or glycosaminoglycan chains using the phenol-sulphuric acid (PS) assay (Additional file 3) and was fractionated by RP-HPLC using a Discovery C18 column (10 mmID × 25 cm) equilibrated with 0.05% TFA and eluted with a linear acetonitrile gradient containing 0.05% TFA at a flow rate of 1.0 mL/min at room temperature. For the PS assay, aliquots from each Sephadex G-15 or Discovery C18 fraction were evaporated and 0.3 mL of water, 0.3 mL of 5% phenol, and 1.5 mL of concentrated sulfuric acid were added. Measuring the absorbance at 490 nm on a spectrophotometer identified the peak containing oligosaccharides.

Eight RP-HPLC fractions (Pr1-d, -e, -h, -i, -j, -k, -l and -p) that were positive for the PS assay (Additional file 3) were digested with chondroitinase ABC as described above. At the end of the incubation period, three volumes of ice-cold ethanol were added to the reaction mixture, which was then centrifuged for 10 min at 10,000 × g. Both supernatant and pellet were lyophilized and stored at -80°C. The lyophilized supernatant was dissolved in 0.6 mL of water and half of the sample was used for the PS assay. The rest of the sample was used for characterization of chondroitin sulfate chains. Two PS negative samples (Pr-1k and l) and five PS positive samples (Pr1-d, -h, -i, -j and -p) were rechromatographed by RP-HPLC and characterized by Edman sequencing.

### Characterization of LMW Dsp in the A extract

The AS2R-g (0.5 mg) fraction containing LMW Dsp (Additional file 4) was dissolved with 1 mL of 0.1 M ammonium bicarbonate/1 mM EDTA-2Na buffer (pH 7.8). Samples were digested by endoproteinase Glu-C (Roche Diagnostics, Indianapolis, IN) using an enzyme/substrate ratio of 1/25 (w/w) at 35°C for 24 h. The digests were applied onto a Discovery C-18 (10 mm × 25 cm) column and run at a flow rate of 1.0 mL/min and monitored at 220 nm (Buffer A: 0.05% TFA; Buffer B: 80% acetonitrile/buffer A). Fifteen fractions were collected, lyophilized, and rechromatographed over the same column.

The 15 purified Glu-C digestion products (approximately 50-100 μg each) in 0.1 M citrate-phosphate buffer (pH 5.0) were incubated with 0.2 mU of glycopeptidase A (Associate of Cape Cod, East Falmouth, MA, USA) containing the Protease Inhibitor Cocktail Set II (0.08 mM of AEBSF, 6.8 μM of Bestatin, 0.8 μM of E-64, 0.35 mM of EDTA and 8 μM of Pepstatin A; Calbiochem, San Diego, CA, USA) at 37°C for 48 h. Three volumes of ice-cold ethanol were added, then the reaction mixture was centrifuged for 10 min at 10,000 × g. Glycopeptides among the Glu-C digestion products were identified by rechromatography using the same Discovery C-18 (10 mm × 25 cm) column. Seven glycopeptides that showed a shift in their retention times following the glycopeptidase A digestion were characterized by LC/MSMS analysis (Nextgen Sciences, Ann Arbor, MI).

### Qualitative and quantitative analyses of sialic acid in Dspp-derived proteins

N-glycosylations were enzymatically released from purified Dpp (ANS1/2-R2) (190 μg), Dsp (ANS1/2R3-R5) (170-250 μg), Dgp (AS2Rd-Rf) (160 μg), and LMW Dsp (AS2R-g) (180 μg) with 1 mU of glycopeptidase A. The porcine 32-kDa enamelin (90 μg) was also digested as a positive control. Three volumes of ice-cold ethanol were added to precipitate the deglycosylated proteins, which were separated from the released N-glycosylations by centrifugation for 10 min at 10,000 × g. N-glycosylations in the supernatants were evaporated and labeled with 2-aminobenzoic acid (2-AA) using 2-AA labeling kit and S Cartridge (QA-bio, Palm Desert, CA). A labeled N-glycan standard (10 pmol; A2 glycan; QA-bio) and the 2-AA-glycosylations were separated by size exclusion-HPLC (SE-HPLC) using a Nanofilm-SEC 150 (7.8 mm × 30 cm) (Sepax Technologies Inc., Newark, DE) column equilibrated with PBS and was eluted with the same solution at a flow rate of 1.0 mL/min at the room temperature. The effluent was continuously monitored by a fluorescence monitor (FP-2020, JASCO, Tokyo, Japan) using an excitation wavelength of 320 nm and an emission wavelength of 420 nm (Additional file 5). The amount of released labeled glycosylations was determined by comparing the area under its chromatographic peak with the labeled A2 glycan standard.

For the qualitative analysis of sialic acid, each of the purified 2-AA glycosylation samples in 50 mM sodium phosphate buffer (pH 6.0) was incubated with 10 mU of sialidase Au (QA-bio) for 1 h at 37°C. An aliquot of the released sialic acid was labeled with 1,2-diamino-4,5-methylenedioxy-benzene (DMB) using a sialic acid labeling kit (QA-bio). The DMB-sialic acid samples were separated by RP-HPLC using a GlycoSep R (2.1 mm × 15 cm) (ProZyme Inc., Hayward, CA) column equilibrated with a mixture of acetonitrile-methanol-water (9:7:84, v/v) and eluted with the same solution at a flow rate of 1.0 mL/min at the room temperature. A fluorescence monitor using an excitation wavelength of 373 nm and an emission wavelength of 448 nm continuously monitored the effluent. The form of sialic acid in each sample was identified by comparing with retention times of a DMB-labeled sialic acid reference panel (QA-bio) (Additional file 5).

For the quantitative analysis of sialic acid we used the SialiQuant sialic acid quantitation kit (QA-bio). LMW Dsp (AS2R-g), HMW Dsp (ANS1/2-R5), and bovine fetuin (33.6 nmol) were digested with sialidase AU. These reactions, as well as 10 nmol of N-acetylneuraminic acid, were digested with N-acetylneuraminic acid aldolase in Tris reaction buffer at 37°C for 30 min. A solution of β-NADH was added and the initial β-NADH absorbance was read at 340 nm (A_340 _initial). The reaction mixture was incubated with lactic dehydrogenase for 1 h at 37°C, then the final β-NADH absorbance was read (A_340 _final) and the nmoles of sialic acid calculated: nmoles of sialic acid = (A_340 _initial - A_340 _final) × 1000/6.22 (mM extinction coefficient of β-NADH is 6.22 at 340 nm) (Additional file 5). The result for the 10 nmol of N-acetylneuraminic acid was 8.68 nmol, so the results of other samples were corrected by multiplying by 1.15. For the bovine fetuin control this correction gave a result of 32.42 nmol.

### SDS-PAGE and Western blotting

SDS-PAGE was performed using Novex 4-20% Tris-Glycine Gel (Invitrogen, Carlsbad, CA, USA). Samples were dissolved in Laemmli sample buffer (Bio-Rad) and electrophoresis was carried out using a current of 30 mA for about 1 h. The gels were stained with Simply Blue Safe Stain (Invitrogen) or Stains-all (Sigma, St. Louis, MO, USA). The apparent molecular weights of protein bands were estimated by comparison with SeeBlue^® ^Plus2 Pre-Stained Standard (Invitrogen). Proteins were electrotransferred from SDS-PAGE onto a Hybond-ECL nitrocellulose membrane (GE Healthcare Bio-Sciences Corp.). Porcine Dsp and mouse chondroitin 6-sulfate (ΔCh-6S) antibodies were used at dilution of 1:50,000. The membrane was immunostained by chemiluminescent detection with ECL Advance Western Blotting Detection Kit (GE Healthcare Bio-Sciences Corp.).

### Amino Acid Analysis

The purified Dsp samples (ANS1/2-R3 to R5) (20-30 μg) were hydrolyzed with 6 N HCl at 115°C for 16 h. The amino acid analyses were performed using a Hitachi L-8900PH instrument at the W. M. Keck Biotechnology Resource Lab at Yale University.

### Automated Edman Degradation

Automated Edman degradation used the 494HT ABI Edman Protein Sequencer at the W.M. Keck Biotechnology Resource Lab at Yale University.

## List of Abbreviations Used

A: Acid extract; AN: acid-salt extract; Bmp: bone morphogenetic protein; A2 glycan: disialo-galactosylated biantennary oligosaccharide; ΔDi-0S: 2-acetamido-2-deoxy-3-O-(beta-D-gluco-4-enepyranosyluronic acid)-D-galactose; ΔDi-4S: 2-acetamido-2-deoxy-3-O-(beta-D-gluco-4-enepyranosyluronic acid)-4-O-sulfo-D-galactose; ΔDi-6S: 2-acetamido-2-deoxy-3-O-(beta-D-gluco-4-enepyranosyluronic acid)-6-O-sulfo-D-galactose; ΔDi-diS_D_: 2-acetamide-2-deoxy-3-*O*-(2-*O*-sulfo-b-D-gluco-4-enepyranosyluronic acid)-6-*O*-sulfo-D-galactose; ΔDi-diS_E_: 2-acetamide-2-deoxy-3-*O*-(b-D-gluco-4-enepyranosyluronic acid)-4,6-bis-*O*-sulfo-D-galactose; ΔDi-triS: 2-acetamido-2-deoxy-3-*O*-(2-*O*-sulfo-b-d-gluco-4-enepyranosyluronic acid)-4,6-di-*O*-sulfo-d-galactose; DD: dentin dysplasia; DGI: dentinogenesis imperfecta; Dgp: dentin glycoprotein; DMB: 1,2-diamino-4,5-methyleneoxybenzene; Dmp1: dentin matrix protein 1; Dsp: dentin sialoprotein; Dspp: dentin sialophosphoprotein; G1: first guanidine extract; G2P: second guanidine pellet; G2: second guanidine extract. GAG: glycosaminoglycan; LMW: low molecular weight; HMW: high molecular weight; HPLC: high performance liquid chromatography; Mmp: matrix metalloproteinase; β-NADH: beta-nicotinamide adenine dinucleotide. NP-HPLC: normal phase-high performance liquid chromatography; Pr: pronase size exclusion fraction; PS: phenol-sulphuric acid; RIM: Residual insoluble material; R: RP-HPLC fraction; RP-HPLC: reversed phase-high performance liquid chromatography; S: size exclusion fraction; SDS-PAGE: sodium dodecyl sulfate-polyacrylamide gel electrophoresis; SCPP: secretory calcium-binding phosphoprotein; SE-HPLC: size exclusion-high performance liquid chromatography; SIBLINGs: small integrin-binding ligand, N-linked glycoproteins; TN: tris-salt extract; 2-AA: 2-aminobenzoic acid.

## Authors' contributions

All authors (YY, TN, JH, FY and JPS) made substantive intellectual contributions to the study, were involved in drafting and revising the manuscript, and have given final approval of the version to be published.

## Supplementary Material

Additional file 1 **Characterization of porcine dentin powder extracts by SDS-PAGE and Western blotting**. This file shows a CBB and a stains-all SDS-PAGE, and a Western blot using a Dsp polyclonal antibody of porcine dentin power extracts and fractions. Click here for file

Additional file 2 **Purification of high molecular weight (HMW) pieces of Dspp that contained Dsp**. This file shows RP-HPLC chromatograms that yielded the ANS1/2-R1 through ANS1/2-R5 fractions and shows these fractions characterized by CBB and stains-all stained SDS-PAGE and Western blotting using a Dsp polyclonal antibody and a Dgp anti-peptide antibody. Click here for file

Additional file 3 **Isolation of glycosylated peptides from the pronase digestion of ANS1/2-R3**. This file shows the size exclusion chromatogram of the A extract and characterization of its three major fractions by CBB and stains-all stained SDS-PAGE, and by Western blotting using Dsp polyclonal antibody. It also shows the RP-HPLC chromatogram for separation of the second size exclusion fraction and characterization of the resulting 14 fractions by CBB and stains-all stained SDS-PAGE, and by Western blotting using a Dsp polyclonal antibody. Click here for file

Additional file 4 **Isolation of low molecular weight (LMW) Dsp components in the A extract**. This file shows the size exclusion chromatogram of pronase-digested ANS1/2-R3 reacted with phenol-sulfate (to detect glycosylations); the RP-HPLC chromatogram that fractionated the potentially glycosylated pronase digestion products into 26 parts; and the results of a spectrophotometric analysis that identified the 8 fractions containing glycosylated peptides. Click here for file

Additional file 5 **Characterization of sialic acid in Dsp N-glycosylations**. This file shows the fluorescent chromatograms from size exclusion-HPLC separations of N-glycosylations that were previously released from various Dsp fractions by glycopeptidase A digestion and then labeled with 2-AA to determine the quantity of their labeled glycosylations by comparing the areas of their chromatographic peaks to those generated by known quantities of a 2-AA labeled standard. Fluorescent chromatograms from the RP-HPLC separations of sialic acid released from the N-glycosylations by sialidase digestion and labeled with DMB are shown that, by comparison to sialic acid standards, allowed the determination of the form of sialic acid on Dsp glycosylations (N-acetylneuraminic acid). Click here for file

Additional file 6 **Alignment of Dsp amino acid sequences from GenBank**. This file shows the amino acid alignment of Dsp from pig, panda, dog, monkey, human, mouse and rat, highlighting the conservation of modified sequences. Click here for file
