# Supplementary material for: Porcine dentin sialoprotein glycosylation and glycosaminoglycan attachments
Source: BMC Biochem. 2011 Feb 3;12:6. doi: 10.1186/1471-2091-12-6 (PMC3039539; doi:10.1186/1471-2091-12-6)
Supplement: Additional file 1 — Characterization of porcine dentin powder extracts by SDS-PAGE and Western blotting. This file shows a CBB and a stains-all SDS-PAGE, and a Western blot using a Dsp polyclonal antibody of porcine dentin power extracts and fractions. [file 1471-2091-12-6-S1.PPT]

## Slide 1
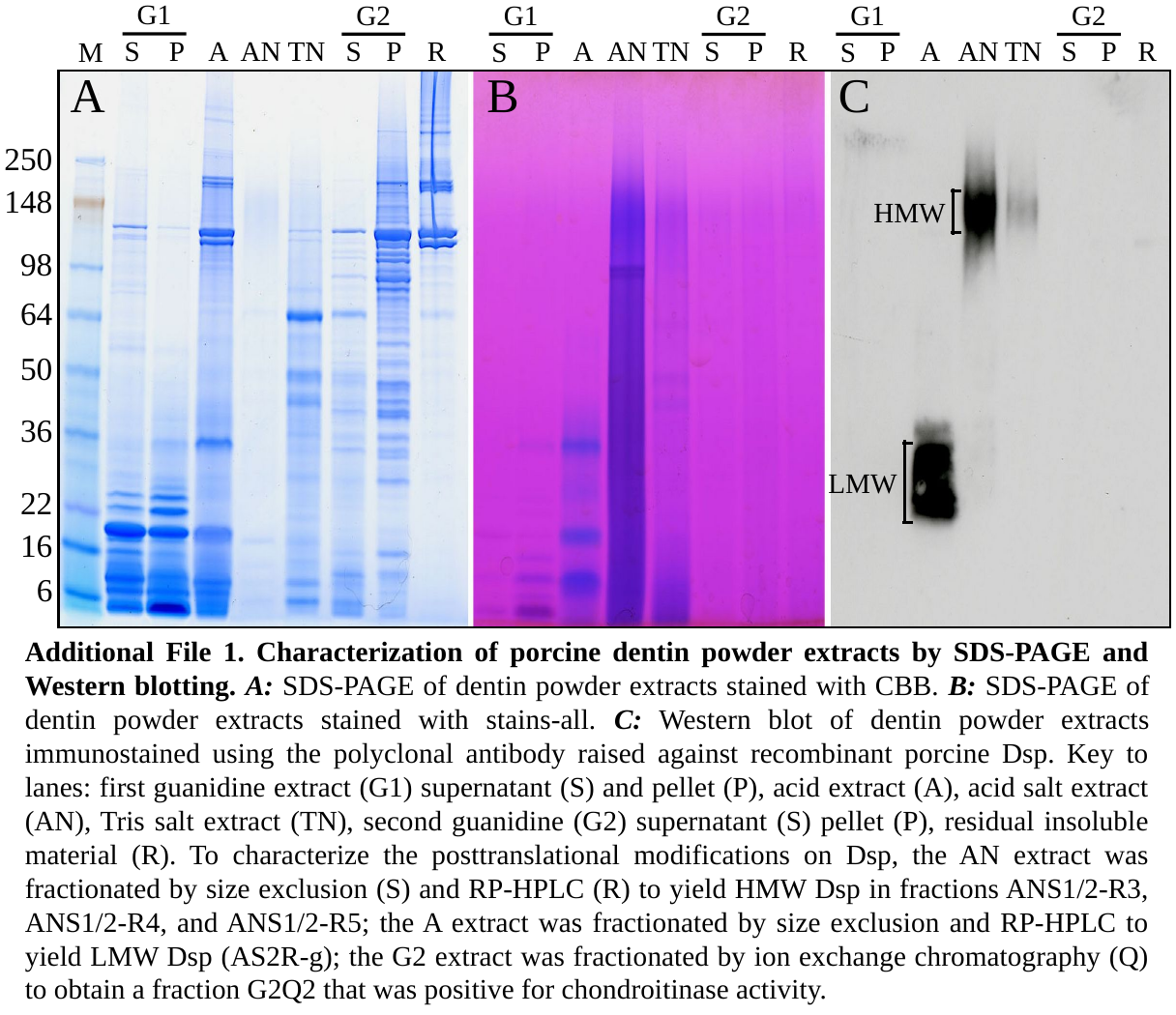

G1
G2
G1
G1
G2
G2
P
S
A
AN
TN
S
P
R
P
S
A
AN
TN
S
P
R
P
S
A
AN
TN
S
P
R
M
A
B
C
250
148
HMW
98
64
50
36
LMW
22
16
6
Additional File 1. Characterization of porcine dentin powder extracts by SDS-PAGE and Western blotting. A: SDS-PAGE of dentin powder extracts stained with CBB. B: SDS-PAGE of dentin powder extracts stained with stains-all. C: Western blot of dentin powder extracts immunostained using the polyclonal antibody raised against recombinant porcine Dsp. Key to lanes: first guanidine extract (G1) supernatant (S) and pellet (P), acid extract (A), acid salt extract (AN), Tris salt extract (TN), second guanidine (G2) supernatant (S) pellet (P), residual insoluble material (R). To characterize the posttranslational modifications on Dsp, the AN extract was fractionated by size exclusion (S) and RP-HPLC (R) to yield HMW Dsp in fractions ANS1/2-R3, ANS1/2-R4, and ANS1/2-R5; the A extract was fractionated by size exclusion and RP-HPLC to yield LMW Dsp (AS2R-g); the G2 extract was fractionated by ion exchange chromatography (Q) to obtain a fraction G2Q2 that was positive for chondroitinase activity.
