# Supplementary material for: Porcine dentin sialoprotein glycosylation and glycosaminoglycan attachments
Source: BMC Biochem. 2011 Feb 3;12:6. doi: 10.1186/1471-2091-12-6 (PMC3039539; doi:10.1186/1471-2091-12-6)
Supplement: Additional file 2 — Purification of high molecular weight (HMW) pieces of Dspp that contained Dsp. This file shows RP-HPLC chromatograms that yielded the ANS1/2-R1 through ANS1/2-R5 fractions and shows these fractions characterized by CBB and stains-all stained SDS-PAGE and Western blotting using a Dsp polyclonal antibody and a Dgp anti-peptide antibody. [file 1471-2091-12-6-S2.PPT]

## Slide 1
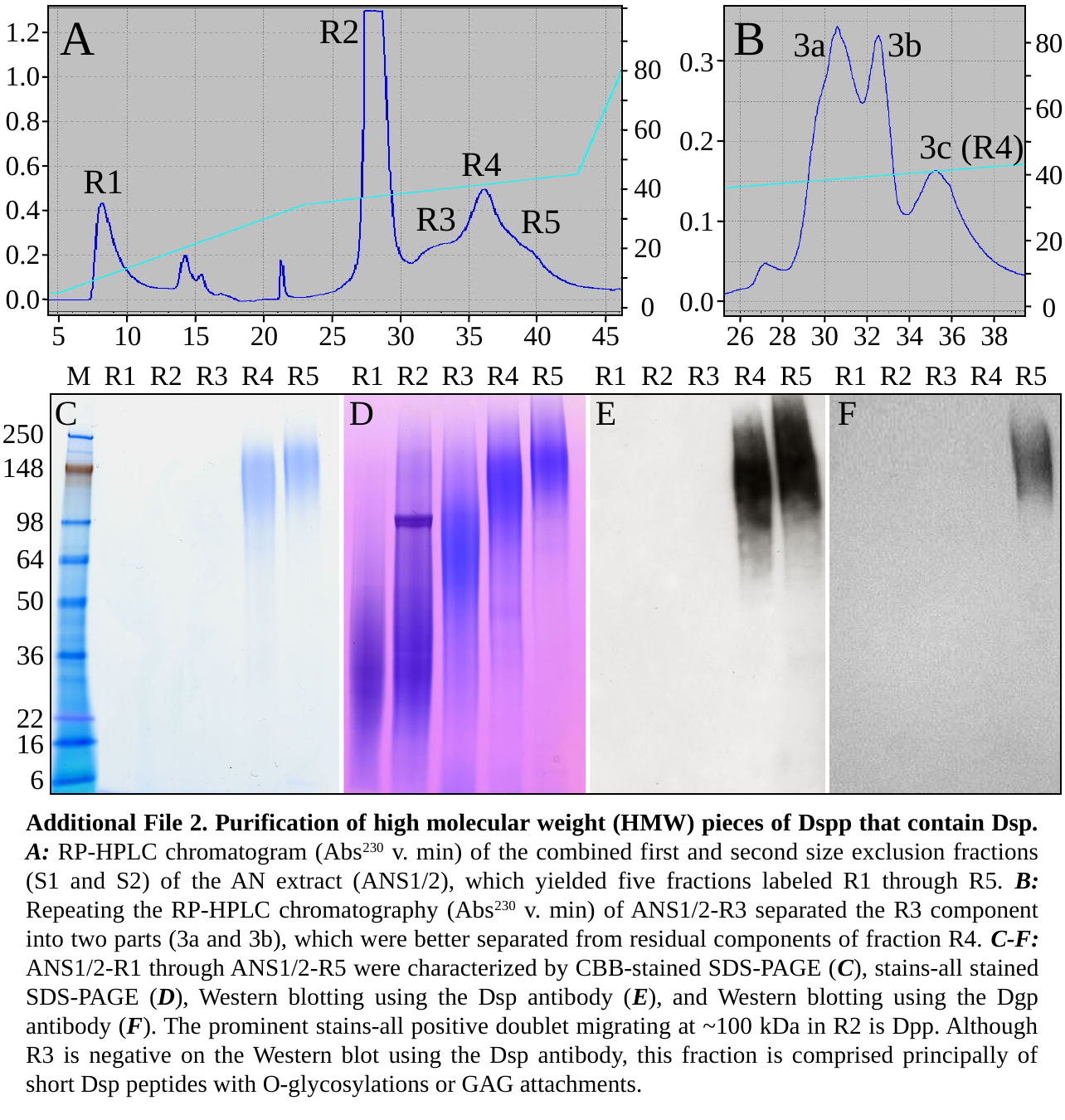

A
B
R2
1.2
3a
3b
80
0.3
80
1.0
60
0.8
60
0.2
3c (R4)
R4
0.6
40
R1
40
0.4
R3
R5
0.1
20
20
0.2
0.0
0.0
0
0
5
10
15
20
25
30
35
40
45
26
28
30
32
34
36
38
M
R1
R2
R3
R4
R5
R1
R2
R3
R4
R5
R1
R2
R3
R4
R5
R1
R2
R3
R4
R5
C
D
E
F
250
148
98
64
50
36
22
16
6
Additional File 2. Purification of high molecular weight (HMW) pieces of Dspp that contain Dsp. A: RP-HPLC chromatogram (Abs230 v. min) of the combined first and second size exclusion fractions (S1 and S2) of the AN extract (ANS1/2), which yielded five fractions labeled R1 through R5. B: Repeating the RP-HPLC chromatography (Abs230 v. min) of ANS1/2-R3 separated the R3 component into two parts (3a and 3b), which were better separated from residual components of fraction R4. C-F: ANS1/2-R1 through ANS1/2-R5 were characterized by CBB-stained SDS-PAGE (C), stains-all stained SDS-PAGE (D), Western blotting using the Dsp antibody (E), and Western blotting using the Dgp antibody (F). The prominent stains-all positive doublet migrating at ~100 kDa in R2 is Dpp. Although R3 is negative on the Western blot using the Dsp antibody, this fraction is comprised principally of short Dsp peptides with O-glycosylations or GAG attachments.
