# Supplementary material for: Porcine dentin sialoprotein glycosylation and glycosaminoglycan attachments
Source: BMC Biochem. 2011 Feb 3;12:6. doi: 10.1186/1471-2091-12-6 (PMC3039539; doi:10.1186/1471-2091-12-6)
Supplement: Additional file 3 — Isolation of glycosylated peptides from the pronase digestion of ANS1/2-R3. This file shows the size exclusion chromatogram of the A extract and characterization of its three major fractions by CBB and stains-all stained SDS-PAGE, and by Western blotting using Dsp polyclonal antibody. It also shows the RP-HPLC chromatogram for separation of the second size exclusion fraction and characterization of the resulting 14 fractions by CBB and stains-all stained SDS-PAGE, and by Western blotting using a Dsp polyclonal antibody. [file 1471-2091-12-6-S3.PPT]

## Slide 1
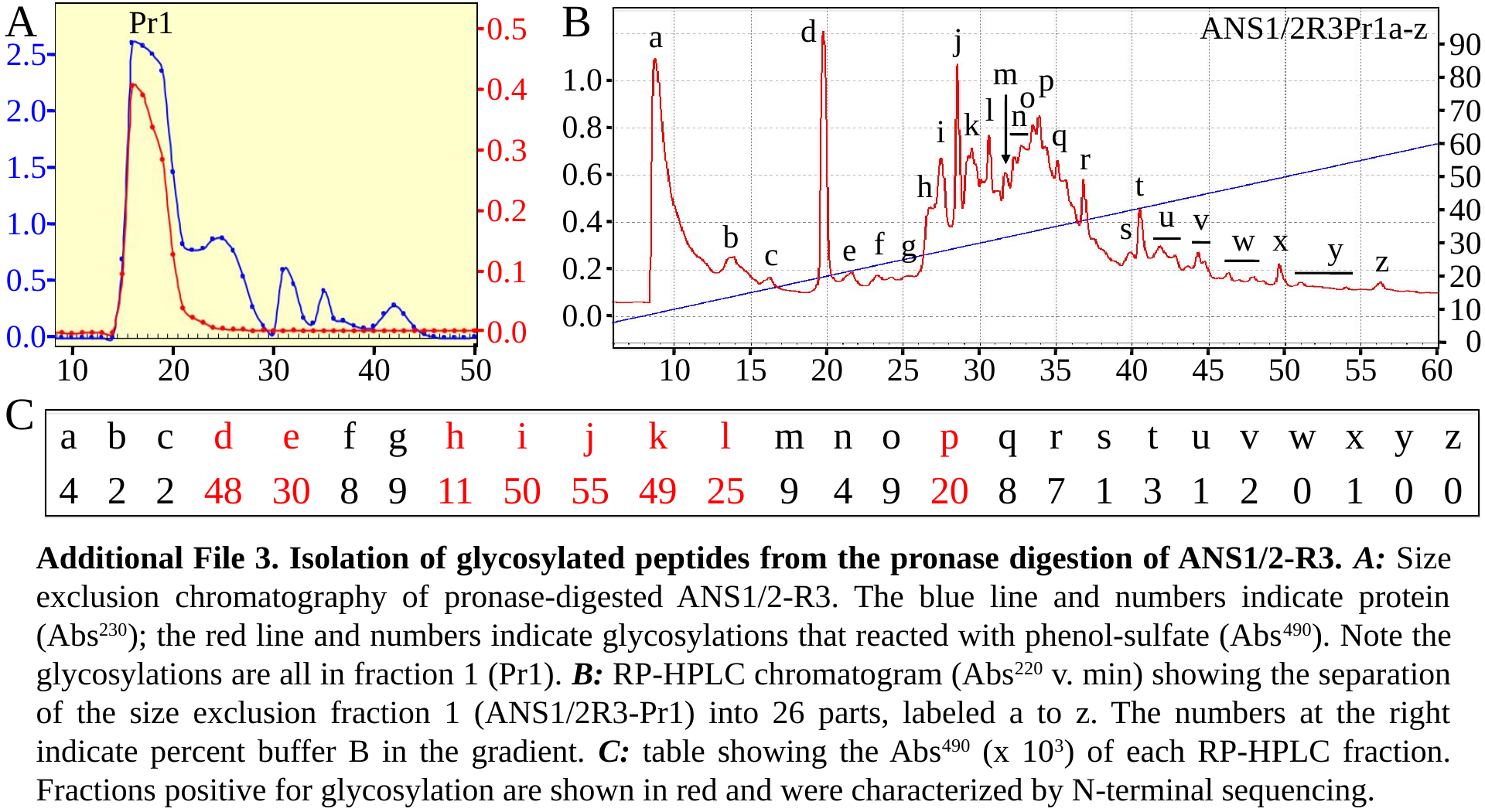

A
B
Pr1
ANS1/2R3Pr1a-z
0.5
d
a
j
90
2.5
m
80
1.0
p
0.4
o
l
70
2.0
n
k
0.8
i
q
60
0.3
r
1.5
0.6
50
t
h
40
0.2
u
v
0.4
1.0
s
b
x
w
30
f
g
y
e
c
z
0.2
0.1
20
0.5
10
0.0
0.0
0.0
0
10
20
30
40
50
10
15
20
25
30
35
40
45
50
55
60
C
a
4
b
2
c
2
d
48
e
30
f
8
g
9
h
11
i
50
j
55
k
49
l
25
m
9
n
4
o
9
p
20
q
8
r
7
s
1
t
3
u
1
v
2
w
0
x
1
y
0
z
0
Additional File 3. Isolation of glycosylated peptides from the pronase digestion of ANS1/2-R3. A: Size exclusion chromatography of pronase-digested ANS1/2-R3. The blue line and numbers indicate protein (Abs230); the red line and numbers indicate glycosylations that reacted with phenol-sulfate (Abs490). Note the glycosylations are all in fraction 1 (Pr1). B: RP-HPLC chromatogram (Abs220 v. min) showing the separation of the size exclusion fraction 1 (ANS1/2R3-Pr1) into 26 parts, labeled a to z. The numbers at the right indicate percent buffer B in the gradient. C: table showing the Abs490 (x 103) of each RP-HPLC fraction. Fractions positive for glycosylation are shown in red and were characterized by N-terminal sequencing.
