# Supplementary material for: Porcine dentin sialoprotein glycosylation and glycosaminoglycan attachments
Source: BMC Biochem. 2011 Feb 3;12:6. doi: 10.1186/1471-2091-12-6 (PMC3039539; doi:10.1186/1471-2091-12-6)
Supplement: Additional file 4 — Isolation of low molecular weight (LMW) Dsp components in the A extract. This file shows the size exclusion chromatogram of pronase-digested ANS1/2-R3 reacted with phenol-sulfate (to detect glycosylations); the RP-HPLC chromatogram that fractionated the potentially glycosylated pronase digestion products into 26 parts; and the results of a spectrophotometric analysis that identified the 8 fractions containing glycosylated peptides. [file 1471-2091-12-6-S4.PPT]

## Slide 1
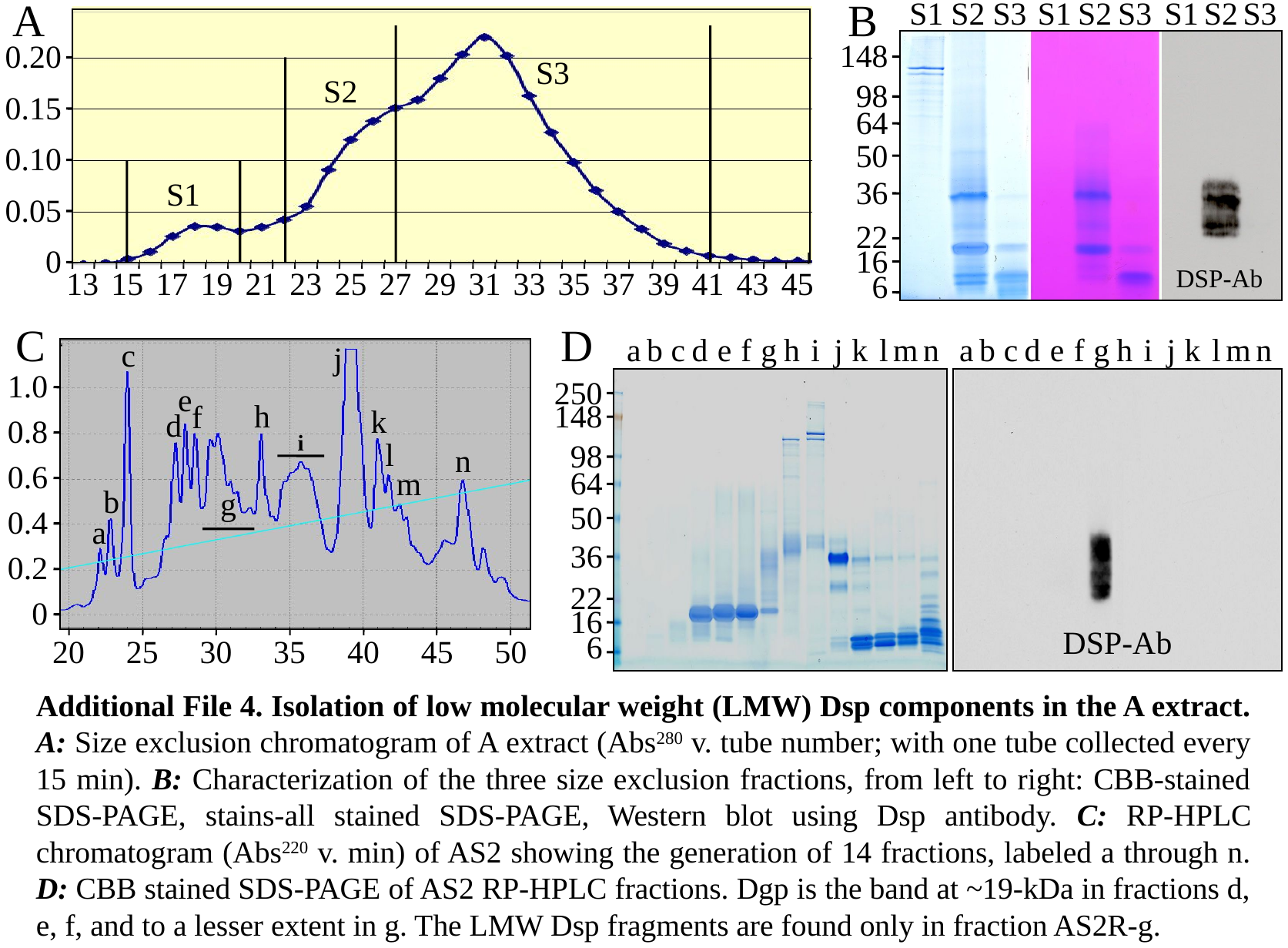

A
B
S1
S2
S3
S1
S2
S3
S1
S2
S3
148
0.20
S3
S2
98
0.15
64
50
0.10
36
S1
0.05
22
16
0
DSP-Ab
13
15
17
19
21
23
25
27
29
31
33
35
37
39
41
43
45
6
C
D
a
b
c
d
e
f
g
h
i
j
k
l
m
n
a
b
c
d
e
f
g
h
i
j
k
l
m
n
c
j
1.0
250
e
h
148
f
k
d
0.8
i
l
98
n
0.6
64
m
b
g
50
0.4
a
36
0.2
22
0
16
DSP-Ab
6
20
25
30
35
40
45
50
Additional File 4. Isolation of low molecular weight (LMW) Dsp components in the A extract. A: Size exclusion chromatogram of A extract (Abs280 v. tube number; with one tube collected every 15 min). B: Characterization of the three size exclusion fractions, from left to right: CBB-stained SDS-PAGE, stains-all stained SDS-PAGE, Western blot using Dsp antibody. C: RP-HPLC chromatogram (Abs220 v. min) of AS2 showing the generation of 14 fractions, labeled a through n. D: CBB stained SDS-PAGE of AS2 RP-HPLC fractions. Dgp is the band at ~19-kDa in fractions d, e, f, and to a lesser extent in g. The LMW Dsp fragments are found only in fraction AS2R-g.
