# Supplementary material for: Porcine dentin sialoprotein glycosylation and glycosaminoglycan attachments
Source: BMC Biochem. 2011 Feb 3;12:6. doi: 10.1186/1471-2091-12-6 (PMC3039539; doi:10.1186/1471-2091-12-6)
Supplement: Additional file 5 — Characterization of sialic acid in Dsp N-glycosylations. This file shows the fluorescent chromatograms from size exclusion-HPLC separations of N-glycosylations that were previously released from various Dsp fractions by glycopeptidase A digestion and then labeled with 2-AA to determine the quantity of their labeled glycosylations by comparing the areas of their chromatographic peaks to those generated by known quantities of a 2-AA labeled standard. Fluorescent chromatograms from the RP-HPLC separations of sialic acid released from the N-glycosylations by sialidase digestion and labeled with DMB are shown that, by comparison to sialic acid standards, allowed the determination of the form of sialic acid on Dsp glycosylations (N-acetylneuraminic acid). [file 1471-2091-12-6-S5.PPT]

## Slide 1
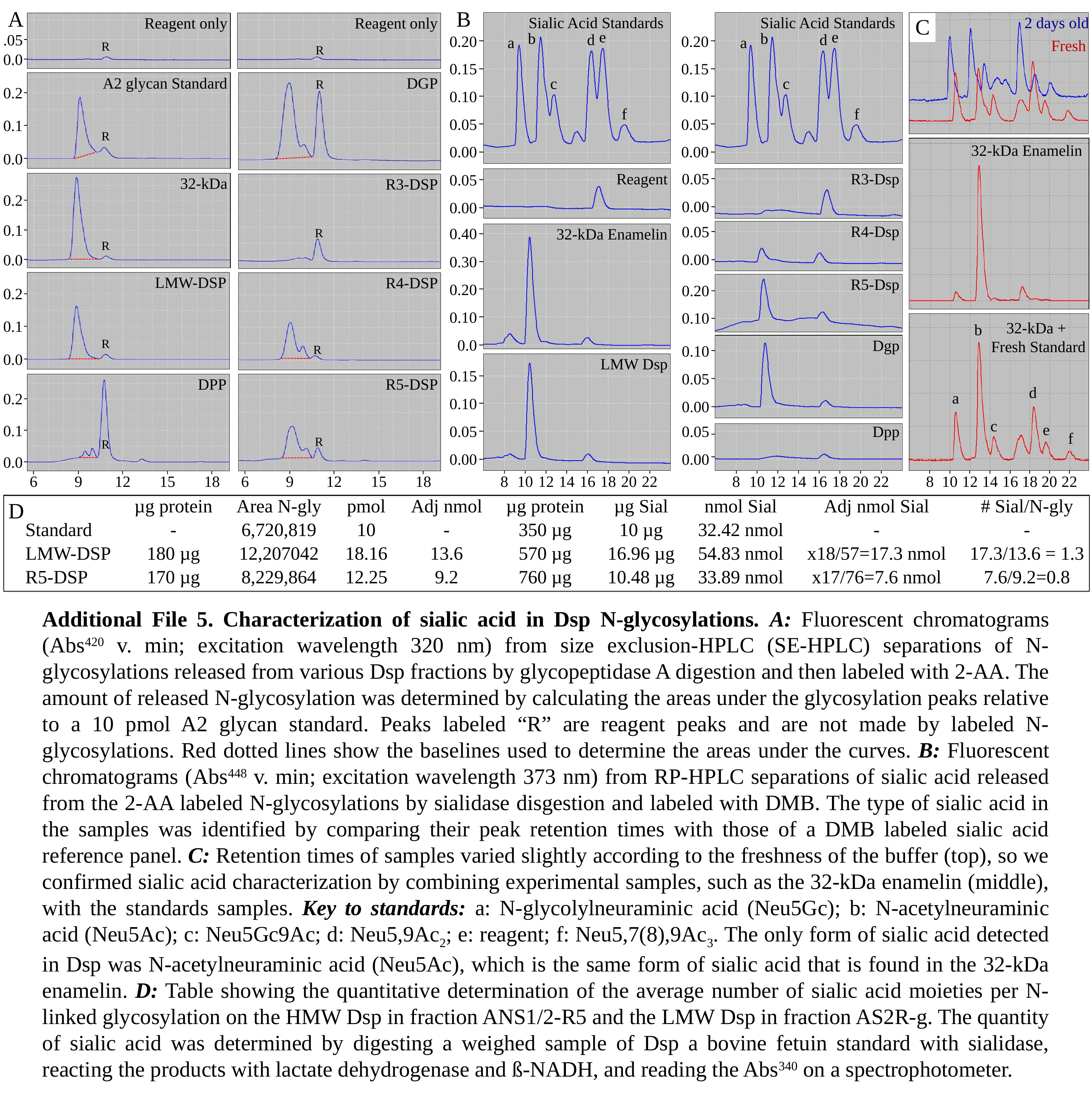

A
Reagent only
Reagent only
.05
R
R
0.0
A2 glycan Standard
DGP
R
0.2
0.1
R
0.0
32-kDa
R3-DSP
0.2
0.1
R
R
0.0
LMW-DSP
R4-DSP
0.2
0.1
R
R
0.0
R5-DSP
DPP
0.2
0.1
R
R
0.0
6
9
12
15
18
6
9
12
15
18
B
Sialic Acid Standards
Sialic Acid Standards
2 days old
C
e
e
b
b
d
d
0.20
0.20
a
a
Fresh
0.15
0.15
c
c
0.10
0.10
f
f
0.05
0.05
32-kDa Enamelin
0.00
0.00
Reagent
R3-Dsp
0.05
0.05
0.00
0.00
R4-Dsp
0.05
0.40
32-kDa Enamelin
0.00
0.30
R5-Dsp
0.20
0.20
0.10
0.10
32-kDa +
Fresh Standard
b
0.0
Dgp
0.10
LMW Dsp
0.15
0.05
d
a
0.10
0.00
c
e
0.05
Dpp
0.05
f
0.00
0.00
8
10
12
14
16
18
20
22
8
10
12
14
16
18
20
22
8
10
12
14
16
18
20
22
µg protein
-
180 µg
170 µg
Area N-gly
6,720,819
12,207042
8,229,864
pmol
10
18.16
12.25
Adj nmol
-
13.6
9.2
µg protein
350 µg
570 µg
760 µg
µg Sial
10 µg
16.96 µg
10.48 µg
nmol Sial
32.42 nmol
54.83 nmol
33.89 nmol
Adj nmol Sial
-
x18/57=17.3 nmol
x17/76=7.6 nmol
# Sial/N-gly
-
17.3/13.6 = 1.3
7.6/9.2=0.8
D
Standard
LMW-DSP
R5-DSP
Additional File 5. Characterization of sialic acid in Dsp N-glycosylations. A: Fluorescent chromatograms (Abs420 v. min; excitation wavelength 320 nm) from size exclusion-HPLC (SE-HPLC) separations of N-glycosylations released from various Dsp fractions by glycopeptidase A digestion and then labeled with 2-AA. The amount of released N-glycosylation was determined by calculating the areas under the glycosylation peaks relative to a 10 pmol A2 glycan standard. Peaks labeled “R” are reagent peaks and are not made by labeled N-glycosylations. Red dotted lines show the baselines used to determine the areas under the curves. B: Fluorescent chromatograms (Abs448 v. min; excitation wavelength 373 nm) from RP-HPLC separations of sialic acid released from the 2-AA labeled N-glycosylations by sialidase disgestion and labeled with DMB. The type of sialic acid in the samples was identified by comparing their peak retention times with those of a DMB labeled sialic acid reference panel. C: Retention times of samples varied slightly according to the freshness of the buffer (top), so we confirmed sialic acid characterization by combining experimental samples, such as the 32-kDa enamelin (middle), with the standards samples. Key to standards: a: N-glycolylneuraminic acid (Neu5Gc); b: N-acetylneuraminic acid (Neu5Ac); c: Neu5Gc9Ac; d: Neu5,9Ac2; e: reagent; f: Neu5,7(8),9Ac3. The only form of sialic acid detected in Dsp was N-acetylneuraminic acid (Neu5Ac), which is the same form of sialic acid that is found in the 32-kDa enamelin. D: Table showing the quantitative determination of the average number of sialic acid moieties per N-linked glycosylation on the HMW Dsp in fraction ANS1/2-R5 and the LMW Dsp in fraction AS2R-g. The quantity of sialic acid was determined by digesting a weighed sample of Dsp a bovine fetuin standard with sialidase, reacting the products with lactate dehydrogenase and ß-NADH, and reading the Abs340 on a spectrophotometer.
