# Supplementary material for: Porcine dentin sialoprotein glycosylation and glycosaminoglycan attachments
Source: BMC Biochem. 2011 Feb 3;12:6. doi: 10.1186/1471-2091-12-6 (PMC3039539; doi:10.1186/1471-2091-12-6)
Supplement: Additional file 6 — Alignment of Dsp amino acid sequences from GenBank. This file shows the amino acid alignment of Dsp from pig, panda, dog, monkey, human, mouse and rat, highlighting the conservation of modified sequences. [file 1471-2091-12-6-S6.PPT]

## Slide 1
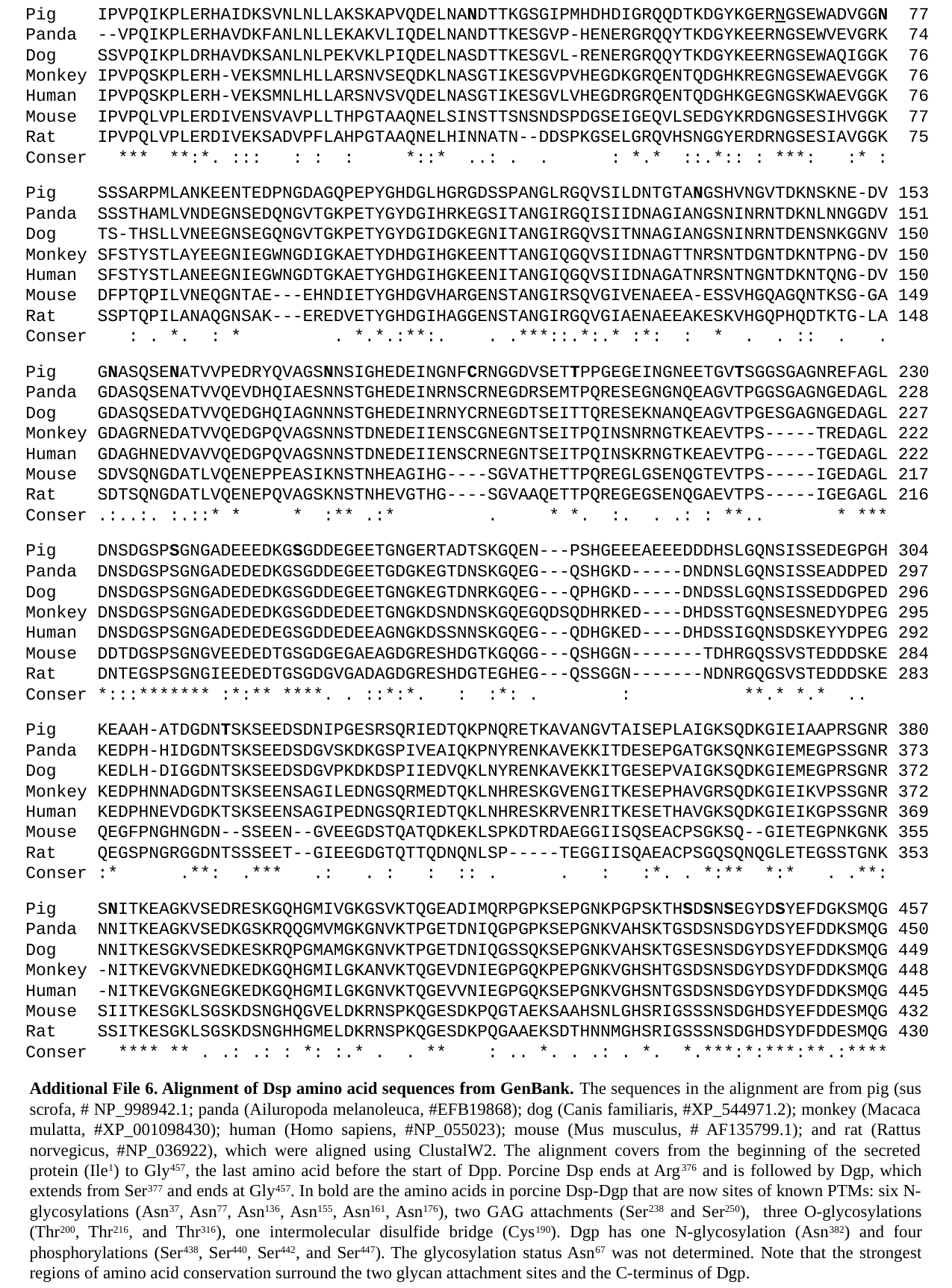

Pig IPVPQIKPLERHAIDKSVNLNLLAKSKAPVQDELNANDTTKGSGIPMHDHDIGRQQDTKDGYKGERNGSEWADVGGN 77
Panda --VPQIKPLERHAVDKFANLNLLEKAKVLIQDELNANDTTKESGVP-HENERGRQQYTKDGYKEERNGSEWVEVGRK 74
Dog SSVPQIKPLDRHAVDKSANLNLPEKVKLPIQDELNASDTTKESGVL-RENERGRQQYTKDGYKEERNGSEWAQIGGK 76
Monkey IPVPQSKPLERH-VEKSMNLHLLARSNVSEQDKLNASGTIKESGVPVHEGDKGRQENTQDGHKREGNGSEWAEVGGK 76
Human IPVPQSKPLERH-VEKSMNLHLLARSNVSVQDELNASGTIKESGVLVHEGDRGRQENTQDGHKGEGNGSKWAEVGGK 76
Mouse IPVPQLVPLERDIVENSVAVPLLTHPGTAAQNELSINSTTSNSNDSPDGSEIGEQVLSEDGYKRDGNGSESIHVGGK 77
Rat IPVPQLVPLERDIVEKSADVPFLAHPGTAAQNELHINNATN--DDSPKGSELGRQVHSNGGYERDRNGSESIAVGGK 75
Conser *** **:*. ::: : : : *::* ..: . . : *.* ::.*:: : ***: :* :
Pig SSSARPMLANKEENTEDPNGDAGQPEPYGHDGLHGRGDSSPANGLRGQVSILDNTGTANGSHVNGVTDKNSKNE-DV 153
Panda SSSTHAMLVNDEGNSEDQNGVTGKPETYGYDGIHRKEGSITANGIRGQISIIDNAGIANGSNINRNTDKNLNNGGDV 151
Dog TS-THSLLVNEEGNSEGQNGVTGKPETYGYDGIDGKEGNITANGIRGQVSITNNAGIANGSNINRNTDENSNKGGNV 150
Monkey SFSTYSTLAYEEGNIEGWNGDIGKAETYDHDGIHGKEENTTANGIQGQVSIIDNAGTTNRSNTDGNTDKNTPNG-DV 150
Human SFSTYSTLANEEGNIEGWNGDTGKAETYGHDGIHGKEENITANGIQGQVSIIDNAGATNRSNTNGNTDKNTQNG-DV 150
Mouse DFPTQPILVNEQGNTAE---EHNDIETYGHDGVHARGENSTANGIRSQVGIVENAEEA-ESSVHGQAGQNTKSG-GA 149
Rat SSPTQPILANAQGNSAK---EREDVETYGHDGIHAGGENSTANGIRGQVGIAENAEEAKESKVHGQPHQDTKTG-LA 148
Conser : . *. : * . *.*.:**:. . .***::.*:.* :*: : * . . :: . .
Pig GNASQSENATVVPEDRYQVAGSNNSIGHEDEINGNFCRNGGDVSETTPPGEGEINGNEETGVTSGGSGAGNREFAGL 230
Panda GDASQSENATVVQEVDHQIAESNNSTGHEDEINRNSCRNEGDRSEMTPQRESEGNGNQEAGVTPGGSGAGNGEDAGL 228
Dog GDASQSEDATVVQEDGHQIAGNNNSTGHEDEINRNYCRNEGDTSEITTQRESEKNANQEAGVTPGESGAGNGEDAGL 227
Monkey GDAGRNEDATVVQEDGPQVAGSNNSTDNEDEIIENSCGNEGNTSEITPQINSNRNGTKEAEVTPS-----TREDAGL 222
Human GDAGHNEDVAVVQEDGPQVAGSNNSTDNEDEIIENSCRNEGNTSEITPQINSKRNGTKEAEVTPG-----TGEDAGL 222
Mouse SDVSQNGDATLVQENEPPEASIKNSTNHEAGIHG----SGVATHETTPQREGLGSENQGTEVTPS-----IGEDAGL 217
Rat SDTSQNGDATLVQENEPQVAGSKNSTNHEVGTHG----SGVAAQETTPQREGEGSENQGAEVTPS-----IGEGAGL 216
Conser .:..:. :.::* * * :** .:* . * *. :. . .: : **.. * ***
Pig DNSDGSPSGNGADEEEDKGSGDDEGEETGNGERTADTSKGQEN---PSHGEEEAEEEDDDHSLGQNSISSEDEGPGH 304
Panda DNSDGSPSGNGADEDEDKGSGDDEGEETGDGKEGTDNSKGQEG---QSHGKD-----DNDNSLGQNSISSEADDPED 297
Dog DNSDGSPSGNGADEDEDKGSGDDEGEETGNGKEGTDNRKGQEG---QPHGKD-----DNDSSLGQNSISSEDDGPED 296
Monkey DNSDGSPSGNGADEDEDKGSGDDEDEETGNGKDSNDNSKGQEGQDSQDHRKED----DHDSSTGQNSESNEDYDPEG 295
Human DNSDGSPSGNGADEDEDEGSGDDEDEEAGNGKDSSNNSKGQEG---QDHGKED----DHDSSIGQNSDSKEYYDPEG 292
Mouse DDTDGSPSGNGVEEDEDTGSGDGEGAEAGDGRESHDGTKGQGG---QSHGGN-------TDHRGQSSVSTEDDDSKE 284
Rat DNTEGSPSGNGIEEDEDTGSGDGVGADAGDGRESHDGTEGHEG---QSSGGN-------NDNRGQGSVSTEDDDSKE 283
Conser *:::******* :*:** ****. . ::*:*. : :*: . : **.* *.* ..
Pig KEAAH-ATDGDNTSKSEEDSDNIPGESRSQRIEDTQKPNQRETKAVANGVTAISEPLAIGKSQDKGIEIAAPRSGNR 380
Panda KEDPH-HIDGDNTSKSEEDSDGVSKDKGSPIVEAIQKPNYRENKAVEKKITDESEPGATGKSQNKGIEMEGPSSGNR 373
Dog KEDLH-DIGGDNTSKSEEDSDGVPKDKDSPIIEDVQKLNYRENKAVEKKITGESEPVAIGKSQDKGIEMEGPRSGNR 372
Monkey KEDPHNNADGDNTSKSEENSAGILEDNGSQRMEDTQKLNHRESKGVENGITKESEPHAVGRSQDKGIEIKVPSSGNR 372
Human KEDPHNEVDGDKTSKSEENSAGIPEDNGSQRIEDTQKLNHRESKRVENRITKESETHAVGKSQDKGIEIKGPSSGNR 369
Mouse QEGFPNGHNGDN--SSEEN--GVEEGDSTQATQDKEKLSPKDTRDAEGGIISQSEACPSGKSQ--GIETEGPNKGNK 355
Rat QEGSPNGRGGDNTSSSEET--GIEEGDGTQTTQDNQNLSP-----TEGGIISQAEACPSGQSQNQGLETEGSSTGNK 353
Conser :* .**: .*** .: . : : :: . . : :*. . *:** *:* . .**:
Pig SNITKEAGKVSEDRESKGQHGMIVGKGSVKTQGEADIMQRPGPKSEPGNKPGPSKTHSDSNSEGYDSYEFDGKSMQG 457
Panda NNITKEAGKVSEDKGSKRQQGMVMGKGNVKTPGETDNIQGPGPKSEPGNKVAHSKTGSDSNSDGYDSYEFDDKSMQG 450
Dog NNITKESGKVSEDKESKRQPGMAMGKGNVKTPGETDNIQGSSQKSEPGNKVAHSKTGSESNSDGYDSYEFDDKSMQG 449
Monkey -NITKEVGKVNEDKEDKGQHGMILGKANVKTQGEVDNIEGPGQKPEPGNKVGHSHTGSDSNSDGYDSYDFDDKSMQG 448
Human -NITKEVGKGNEGKEDKGQHGMILGKGNVKTQGEVVNIEGPGQKSEPGNKVGHSNTGSDSNSDGYDSYDFDDKSMQG 445
Mouse SIITKESGKLSGSKDSNGHQGVELDKRNSPKQGESDKPQGTAEKSAAHSNLGHSRIGSSSNSDGHDSYEFDDESMQG 432
Rat SSITKESGKLSGSKDSNGHHGMELDKRNSPKQGESDKPQGAAEKSDTHNNMGHSRIGSSSNSDGHDSYDFDDESMQG 430
Conser **** ** . .: .: : *: :.* . . ** : .. *. . .: . *. *.***:*:***:**.:****
Additional File 6. Alignment of Dsp amino acid sequences from GenBank. The sequences in the alignment are from pig (sus scrofa, # NP_998942.1; panda (Ailuropoda melanoleuca, #EFB19868); dog (Canis familiaris, #XP_544971.2); monkey (Macaca mulatta, #XP_001098430); human (Homo sapiens, #NP_055023); mouse (Mus musculus, # AF135799.1); and rat (Rattus norvegicus, #NP_036922), which were aligned using ClustalW2. The alignment covers from the beginning of the secreted protein (Ile1) to Gly457, the last amino acid before the start of Dpp. Porcine Dsp ends at Arg376 and is followed by Dgp, which extends from Ser377 and ends at Gly457. In bold are the amino acids in porcine Dsp-Dgp that are now sites of known PTMs: six N-glycosylations (Asn37, Asn77, Asn136, Asn155, Asn161, Asn176), two GAG attachments (Ser238 and Ser250), three O-glycosylations (Thr200, Thr216, and Thr316), one intermolecular disulfide bridge (Cys190). Dgp has one N-glycosylation (Asn382) and four phosphorylations (Ser438, Ser440, Ser442, and Ser447). The glycosylation status Asn67 was not determined. Note that the strongest regions of amino acid conservation surround the two glycan attachment sites and the C-terminus of Dgp.
